# A vector platform for the rapid and efficient engineering of stable complex transgenes

**DOI:** 10.1038/srep34365

**Published:** 2016-10-03

**Authors:** Carsten Jäckel, Melanie Schmitt Nogueira, Nadja Ehni, Christiane Kraus, Julius Ranke, Maike Dohmann, Elfriede Noessner, Peter J. Nelson

**Affiliations:** 1 Medizinische Klinik und Poliklinik IV, Klinikum Innenstadt, Ludwig-Maximilians-University Munich, Clinical Biochemistry Group, Munich, Germany; 2 Helmholtz Zentrum München Immunoanalytics- Core Facility, Großhadern, Germany

## Abstract

We describe the generation of a set of plasmid vector tools that allow the rapid generation of complex-interacting stable transgenes in immortalized and primary cells. Of particular importance is inclusion of a mechanism to monitor the activation status of regulatory pathways via a reporter cassette (using *Gaussia* Luciferase), with control of additional transgene expression through doxycycline de-repression. The resulting vectors can be used to assess regulatory pathway activation and are well suited for regulatory pathway crosstalk studies. The system incorporates MultiSite-Gateway cloning for the rapid generation of vectors allowing flexible choice of promoters and transgenes, and Sleeping Beauty transposase technology for efficient incorporation of multiple transgenes in into host cell DNA. The vectors and a library of compatible Gateway Entry clones are available from the non-profit plasmid repository Addgene.

Individual signaling pathways often undergo cross talk, essentially establishing a regulatory network that drives specific biologic actions^1^,^2^. These networks help define tissue homeostasis and moderate the cellular response to external stimuli. In cancer studies, minor genetic aberrations can lead to fundamentally changed phenotypes by influencing regulatory pathways and networks^3^,^4^,^5^. Typical methods used for the study of regulatory pathways include the overexpression, or knockdown, of specific genes and the monitoring of pathway targets to assess the downstream effects of these treatments. Engineering cells with complex transgenes can help monitor and test the regulatory criteria linked to specific questions associated with different modes of pathway activation such as crosstalk.

The transfer of genetic cargo into experimental cells requires various chemical or physical strategies, many involving the transient introduction of the genetic material. Transient transfection, especially when dealing with reporter cassettes, can be problematic as it induces cell stress, and can show highly variable transfection efficiency with rapid signal degradation. The stable integration of genetic cargo can help circumvent these problems, but the wide scale application of stably introduced constructs can be inefficient and time consuming. Problems with stable transfection can arise due to the low efficiency of the techniques used, low integration copy-numbers, and the potential epigenetic silencing of the transgenes. Viral vector systems can be used to efficiently transfect cells - but these systems are limited by the general viral packaging biology used, including limits on the size and complexity of the transgenes studied. For example, the introduction of additional promoters, splice sites or poly-A signals etc. needed for the efficient control of expression of multiple expression cassettes in the same vector are problematic when dealing with retroviral vectors. Finally, the application of viral systems is complicated by the increased biosafety level required for their use. We describe here the generation of a flexible and efficient plasmid-based vector system for the rapid generation of transgenes in primary cells and cell lines.

Transposon-based systems can allow the efficient integration of genetic cargo into host genomes via an active process catalyzed by a specific transposase. Stable integration of the transgene is achieved by flanking it with inverted terminal repeats (ITR) specifically recognized by a transposase enzyme. For the integration of transgenic material into target genomes, a second transposase-expressing plasmid is transiently co-introduced with the plasmid(s) of interest containing ITR-flanked genetic cargo. The vector system detailed here incorporates Sleeping Beauty transposon (SB) technology. SB-based stable integration was developed by reactivation of an endogenous transposon (belonging to the Tc1/mariner type transposon family) originally identified in salmonid fish^6^. This defective endogenous transposon was re-activated by site directed mutagenesis and was subsequently re-engineered to optimize activity (the SB100X transposase is available from Addgene)^7^. The efficacy of the SB system for the stable transfection of animal cells including primary cells such as T cells, and mesenchymal stem cells, is well established, and importantly, its use does not require higher bio-safety levels^8^,^9^,^10^,^11^,^12^,^13^. An additional advantage of the SB system is that the ITR sequences flanking the genetic cargo have been shown to have an “insulator-like” function, leading to reduced gene-silencing of the integrated transgene by epigenetic processes^14^. The high efficiency of SB-mediated insertion into the host cell genome allows the integration of multiple vector cassettes in the context of a single transfection reaction. One issue that should be considered when using active genomic integration events, is the site specificity of the mechanism used. SB transposons integrate at TA-nucleotides with an estimated 200 million potential integration sites in the human genome, so integration is quasi-random.

More recently, variations of SB compatible expression vectors have been described that further expand the possibilities of the system by offering a platform for either single fragment Gateway cloning or inducible promoters^15^,^16^. The vector system detailed here further expands the SB toolbox by adding a MultiSite Gateway compatible vector family allowing for highly efficient cloning strategies of extremely complex transgenes from up to four fragments in a directional manner, and a second set of stably integratable reporter vectors for monitoring the activation state of signaling pathways. These highly flexible vectors were developed for rapid cloning and engineering of primary cells and cell lines, providing bulk transgenic cell pools that are usable for experiments after only a few passages for recovery and selection. In practice, the frequency of stable integration is limited only by the general efficiency of cell transfection. The inclusion of nucleofection methods for target cell transfection^17^,^18^,^19^ allows a high level of transfection efficiency paired with the facilitated genomic integration of vectors using the SB system.

## Methods

### Cloning

The modification steps in the vector backbones were carried out using restriction enzyme cloning and T4 DNA Ligase as per manufacturer’s instructions. Propagation and transformation of Gateway cloning destination vectors was done in DB3.1 chemically competent *E. coli* (Thermo Fischer). To introduce responsive elements into pSBTET.reporter backbones, custom DNA oligos were designed using previously validated transcription factor binding sites such that upon annealing they generated PstI/XhoI overhangs (the TF binding site sequences used are provided in Supplemental Sequences, and complete vector sequences are available from Addgene). The annealed double-stranded oligos were then ligated into the vector backbone. Gateway reactions were carried out according to manufacturer’s instructions using the MultiSite Gateway Pro Plus Kit (Thermo Fischer). Entry Clones were created using Gateway BP Clonase II Enzymes, Expression clones were generated using Gateway LR Clonase II Plus. All Gateway reactions were transformed into MACH-1 chemically competent *E. coli* (Thermo Fischer).

### Design of pSBTET.reporter

The Invitrogen pCDNA6/TR TET-Repressor expression vector was modified to include Sleeping Beauty inverted terminal repeats (SB ITRs). SB ITRs were mobilized by PCR to include PciI or SgrDI overhangs and introduced into the same cleavage sites into the backbone of pCDNA6/TR yielding pCDNA6/TR-ITR. Stable integration was validated by comparison of pCDNA6/TR with and without co-transfection with the SB100X plasmid. pCDNA6/TR-ITR showed at least an order of magnitude more stable events as compared to the random non-active integration strategy (data not shown). In the next step, a *Gaussia* luciferase based reporter cassette was introduced into pCDNA6/TR-ITR while retaining the TET-Repressor function. The *Gaussia* luciferase gene was mobilized by PCR and introduced between the HindIII and XbaI sites of the pGL3-Promoter vector with a minimal CMV promoter (38 bp length). The *Gaussia* luciferase-driving reporter cassette was then lifted out via PCR and introduced into pCDNA6/TR-ITR between NotI and BstBI restriction sites. This was done in a multiple step process yielding the pSBTET.reporter vector. Further variants of pSBTET.reporter were then generated by inserting validated multimers of transcription factor binding sites between the XhoI and PstI sites in the backbone (see Supplemental Sequences).

### Design of the pSBDEST vector

Single fragment Gateway cloning is a widely used cloning strategy for the specific and high-throughput cloning of complex sequences, but limited by the context of the destination vectors available. A promoterless Gateway destination vector was generated to be compatible with the Sleeping Beauty transposon system, allowing rapid generation of stable transgenes, with the benefit of high flexibility of MultiSite Gateway cloning strategies. The Invitrogen vector pcDNA6.2/V5-PL-DEST was modified to include SB ITRs and three additional versions of the vector with other selection cassettes (Neomycin, Zeocin and Hygromycin) were generated. Finally, for constitutive expression studies, a version of the vector was generated where expression of the gene of interest was linked to expression of the selective marker (Puromycin) via an internal ribosomal entry site (pSBDEST.IP).

### Cells and transfection

The pCMV(CAT)T7-SB100 expression vector was a gift from Zsuzsanna Izsvak (Addgene plasmid # 34879). HEK293 cells were cultured in DMEM + 10% FCS + 1% Penicillin-Streptomycin. HEK293 cells were obtained from ATCC (Manassas, Virginia). HEK293 cells were chosen as they are responsive to wide range of stimuli. Transfection of HEK293 cells was performed using the AMAXA Nucleofector IIb machine (Lonza) using buffer 1 M (5 mM KCl; 15 mM MgCl2; 120 mM Na2HPO4/NaH2PO4 pH7.2; 50 mM Mannitol). Briefly, 1 × 10^6^ HEK293 were resuspended in 100 μl buffer 1 M and transfected with 4 μg of reporter/expression plasmid and 2 μg SB100X vector using program Q-001. Alternatively, a NEON electroporation device can be used for transfection of HEK293 cells resulting in higher efficiency (using 1150 V, 30 ms, 1 pulse in 100 μl NEON-tips).

After transfection, the cells were cultured in 25 cm^2^ flasks. 24 hours post transfection HEK293 cells were selected with either Blasticidin 8 μg/ml, Hygromycin 150 μg/ml, G418 600 μg/ml, Puromycin 3 μg/ml or Zeocin 150 μg/ml. Selection is carried out for 7–10 days, as per antibiotic manufacturer’s instructions. Upon reaching 80–90% confluency, 20,000 HEK293 reporter cells can be seeded into 96 wells with three to five replicates depending on the experiment, in 100 μl total volume of culture medium, and subjected to stimulation.

Primary human bone marrow-derived mesenchymal stem cells (hBMSC) were isolated (from consenting donors in compliance with ethics commission of the LMU Munich) as previously described^20^,^21^ and cultured in DMEM + 10% FCS + 1% Penicillin-Streptomycin + 5% Thrombocyte conserves and 1 μl/ml Heparine (hBMSC medium). Transfections were performed at passage three using the Neon Transfection System (Thermo Fischer Scientific). Four ×10^5^ to 5 × 10^5^ hBMSC were resuspended in 10 μl Buffer R (provided in the Neon Transfection Kit), and transfected with 2 μg of each plasmid containing the transgenes, in combination with 1 μg SB100X transposase expression plasmid, using 2 pulses, 1050 V, 30 ms pulse width, according to H9 Human Embryonic Stem Cell protocol provided for Neon. The cells were then transferred into 25 cm^2^ flasks, and 24 hours later, antibiotic selection was initiated. For reporter constructs, cells were generally selected with Blasticidin 2 mg/ml or Puromycin 0.6 μg/ml. To limit stress to the primary hBMSC, selection was raised step-wise: with 40% of total antibiotic concentration given after 24 h, then brought to full antibiotic selection 72 h after transfection. For constructs containing both a reporter element and an overexpression element, selection was performed using Blasticidin 2 μg/ml in combination with Hygromycin 40 μg/ml. For double selection, antibiotic concentration was raised more carefully: with 25% of each antibiotic given after 24 h, and then increasing antibiotic levels by 25% every 48 h until full selection pressure was reached.

For *Gaussia* luciferase experiments, 8,000–16,000 cells were seeded at least in triplicate per treatment group into 96-well plates, and grown to 70–80% confluency with a total volume of 100 μl medium per well. Medium was changed 24 h after plating, and substituted with DMEM medium without additives. Stimulation was performed 24 h later, and *Gaussia* Luciferase activity measured 24 h and 48 h after stimulation. hBMSCs transfected with pSB.IP.CAG.sGFP were cultured in hBMSC medium and were at passage six at observation point.

In the experiments carried out here, transfections in primary cells were generally performed and maintained as bulk cultures, but single cell cloning can also be performed when the proliferative capacity of the engineered cells is not limited.

All Doxycycline stimulations were carried out using a final concentration of 1 μg/ml Doxycycline.

Human monocytes were isolated from peripheral blood mononuclear cells (taken from consenting donors in compliance with ethics commission of the LMU Munich) using MACS and enriched using CD14 microbeads. Monocytes were polarized to M1, M2 and MCSF phenotypes as previously described^22^. For co-culture experiments, 20,000 Hek293 reporter cells were seeded into 96 well plates. After 2 hours for attachment, 40,000 polarized myeloid cells were added for co-culture.

Cell lines were routinely tested for Mycoplasma infection using the MycoAlert™ PLUS Mycoplasma Detection Kit (Lonza, Basel).

### Luciferase reporter assays

Twentyfour and 48 hours post stimulation, luciferase activity was assayed by removing 20 μl of conditioned growth media from each well. Luciferase activity was measured using the BioLux *Gaussia* Luciferase Assay Kit in a Berthold Lumat LB 9507 Luminometer with 10 s measurements. Briefly, 20 μl cell culture supernatant were mixed with 50 μl *Gaussia* luciferase assay solution, allowed to incubate for 45 s and measured. All experiments were performed in three to five replicates to allow for assessment of significance. Data from experiments was analyzed using Graphpad Prism Software (GraphPad Software, LaJolla).

## Results and Discussion

**Figure 1:**
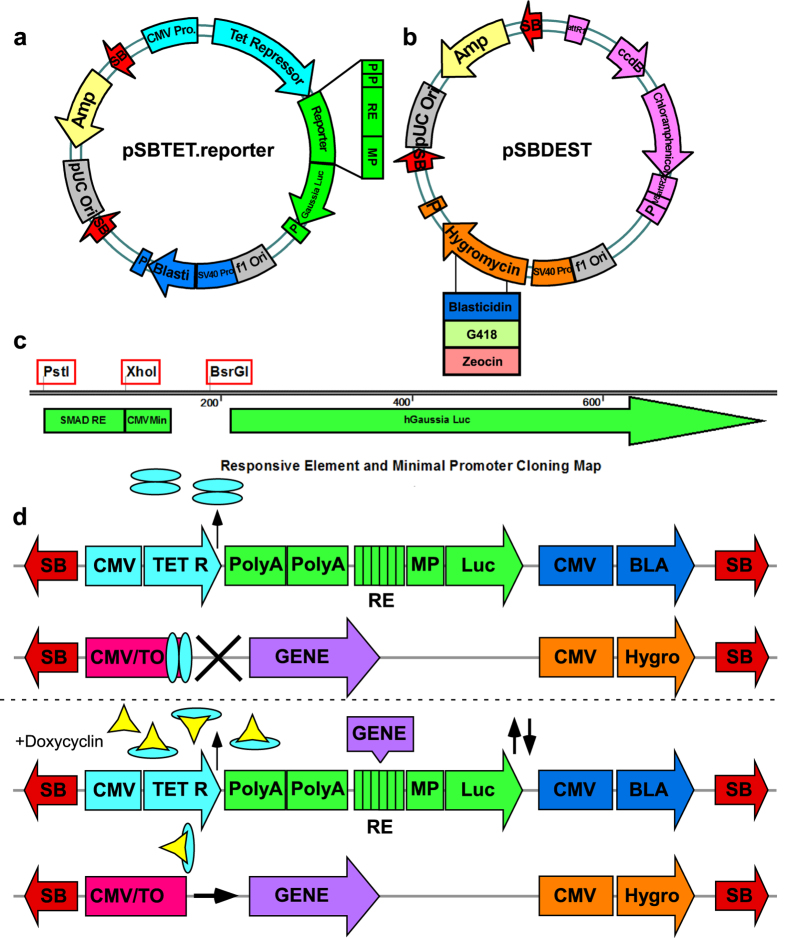
Overview of vector platform. (**a**) Map of the pSBTR.reporter vector. Sleeping Beauty (SB) ITRs, P: poly-adenylation signal sequence, RE: transcription factor responsive element, MP: minimal promoter. (**b**) Map of the pSBDEST vector family. (**c**) Map of accessible restriction sites in the reporter cassette. (**d**) Functional diagram of reporter modulation with an inducible expression vector. The vector platform allows inducible overexpression/knockdown of genes from two stably integrated vectors. The first vector carries constitutive expression of the Tet-Repressor gene, a luciferase reporter cassette and Blasticidin selection. The second vector can be used for Gateway engineered and doxycycline inducible overexpression constructs, with a series of available selective markers as exemplified here by Hygromycin. The vectors can be stably integrated in parallel by co-transfection with the SB100X transposase vector, or engineered in series.

The vector system described here is based on two vector families, members of each can be introduced in parallel, or sequentially into cells of interest. The pSBTR.reporter (Fig. 1a,c) is derived from the commercial pcDNA6/TR vector, which was modified to include SB ITRs and a pathway reporter cassette driving secreted *Gaussia* Luciferase^23^. The pSBTR.reporter vectors constitutively express the TET-Repressor protein which is used to control the activity of TET-operator-inducible promoters on a second vector. The *Gaussia* luciferase reporter is secreted, and can be sampled directly from the growth media without cell lysis, thus allowing time course experiments. The system is amenable for high throughput formats. A series of variants of the reporter vector based on multimers of select transcription factor (TF) binding sites have been validated for the measurement of different regulatory pathways (Table 1).

The pSBDEST (Fig. 1b) vector is represented by a family of promoterless MultiSite-Gateway destination vectors based originally on the pcDNA6.2/V5-PL-Dest vector, but modified by the introduction of SB ITRs, and additional selection markers (available with four different selective markers). The MultiSite Gateway cloning platform allows for the rapid (one step) generation of complex promoter and transgene combinations based on up to four fragment recombination (see Supplemental Fig. S1). An additional variant of the vector includes an internal Ribosomal Entry Site (IRES) driving Puromycin resistance downstream of the Gateway cloning cassette allowing the expression of a transgene linked to the selective marker (Table 1). A series of compatible entry clones usable in conjunction with the expression vectors have been deposited at Addgene (Table 2). Among these is the pENTR221-CMV/TO. Pro vector, an entry clone carrying the Tet-Operator controlled CMV promoter. When used together with any of the pSBTR.reporter vectors, the constitutively expressed TET-repressor protein blocks transcription from the Tet-Operator controlled promoter^24^. CMV/TO is de-repressed by addition of Doxycycline (Fig. 1d) allowing inducible transgene expression with the parallel monitoring of pathway activation via the appropriate pathway reporter.

**Figure 2:**
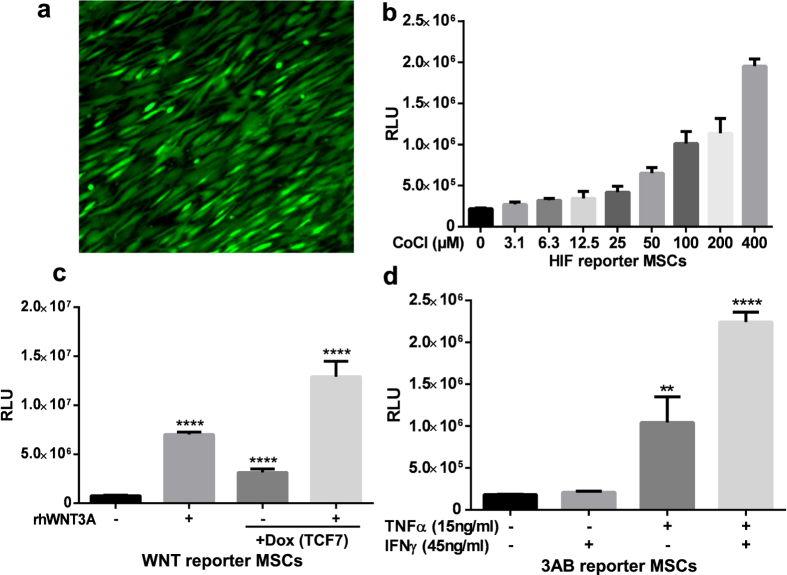
Sample hBMSC data generated with this vector system (**a**) Primary human bone marrow-derived MSCs were transfected with pSB.IP.CAG.sGFP, a constitutive sGFP expression plasmid after 14 days selection and recovery. (**b**) hBMSCs transfected with pSBTR.Hypoxia were seeded in duplicates and subjected to increasing levels of CoCl_2_, an agent inducing a hypoxia-like reaction. 48 hours post stimulation *Gaussia* luciferase activity was assayed. (**c**) hBMSCs were stably transfected with the pSBTR.WNT reporter plasmid, and pSB.H.CMV/TO.TCF7, a doxycycline inducible TCF7 expression vector. Stimulation with doxycycline (1 μg/ml) or control recombinant human WNT3A (400 ng/ml) protein leads to activation of the reporter construct. Treatment with both WNT3A and doxycycline increases the effect. (n = 3 per group, experiment repeated three times) (**d**) hBMSCs were stably transfected with pSBTR.3x (RaPM), a construct based on a multimer of a promoter module found in the human CCL5 promoter. Cells were treated with either 15 ng/ml recombinant human TNFα, 45 ng/ml recombinant human IFNγ or both for 48 hours and *Gaussia* luciferase activity was assayed. TNFα alone activates the reporter while IFNγ alone does not. TNFα and IFNγ in combination leads to more than additive effect. (**** Significances where indicated p < 0.0001, ***p < 0.001, **P < 0.01, n.s. = not significant, based on unpaired t-test; bars are mean with SD).

**Figure 3:**
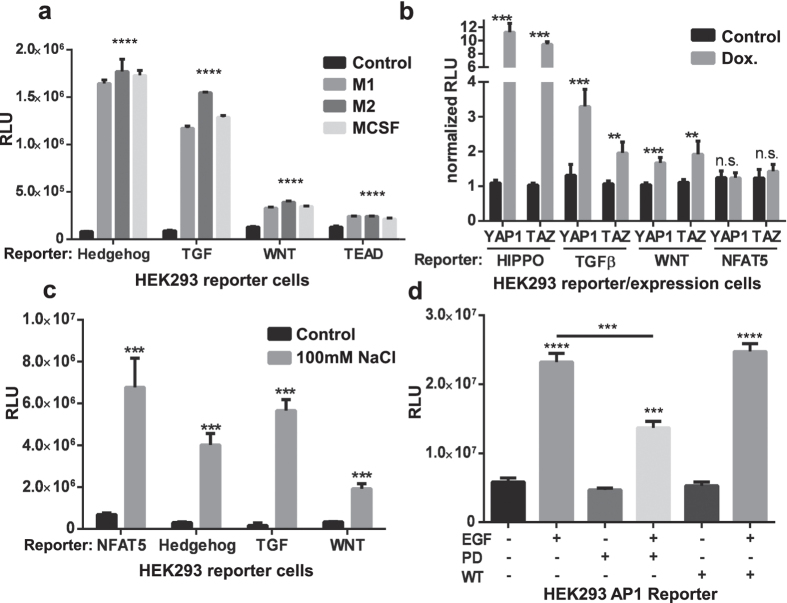
Co-culture and inhibition data generated with stable HEK293 reporter cell lines. (**a**) Co-culture of HEK293 reporter cells with primary human M1, M2-polarized or MCSF unpolarized macrophage cells (20,000 HEK293 cells seeded out with 40,000 myeloid cells and co-incubated for 48 hours. *Gaussia* luciferase reporter activity in HEK293 Reporter cells was measured for Hedgehog (GLI), TGF-β (SMAD), WNT (TCF/LEF) and Hippo (TEAD) Pathways. Co-culture strongly activates three of the pathways, and to a much lesser degree Hippo signaling. (n = 3 per group, experiment repeated three times) (**b**) Crosstalk study between pathways. HEK293 cells were transfected with pSBTR.reporter vectors for either Hippo (TEAD), WNT (TCF/LEF), TGF-β (SMAD) or NFAT5 pathways, and Doxycycline inducible, constitutively active mutant expression constructs for human YAP1 or TAZ (also known as WWTR1) - transcriptional co-activators of the Hippo signaling pathway. Upon induction with Doxycycline (1 μg/ml), activation of the canonical Hippo and potential target pathways was measured. Values are normalized on lowest value per experiment. (n = 4 per group, experiment repeated twice) (**c**) Assessment of the effect of osmotic stress on HEK293 reporter cell lines. Hek293 reporter cell lines for NFAT5, a classical osmotic responsive transcription factor, Hedgehog (GLI), TGF-β (SMAD), and WNT (TCF/LEF) were cultured either in normal medium or in medium supplemented with 100 mM NaCl. 48 hours post stimulation *Gaussia* luciferase activity was assayed. High salt concentration strongly and unspecifically activates all the assessed reporters. (**d**) Hek293 AP1 reporter cells were either stimulated with 20 ng/ml Epithelial Growth Factor recombinant protein (EGF), 25 μM MAPK inhibitor PD98059 or 200 nM PI3K inhibitor Wortmannin (WT). 48 hours post stimulation *Gaussia* luciferase activity was assayed. PD98059 strongly reduces the efficacy of EGF activation while Wortmannin does not. (**** Significances where indicated p < 0.0001, ***p < 0.001, **P < 0.01, n.s. = not significant, based on unpaired t-test; bars are mean with SD).

To help demonstrate the potential application of this vector system, a series of examples are provided in Figs 2 and 3. Figure 2a shows primary human mesenchymal stem cells engineered with a vector based on pSBDEST.IP, a Gateway Destination vector carrying an IRES downstream of the Gateway acceptor cassette. The resulting pSB.IP.CAG.sGFP (7516 bp, SB cassette 5660 bp) expression vector provides constitutive sGFP expression (driven by the synthetic CAG Promoter) linked to antibiotic selection^25^. Similar studies have reported integration numbers that range from 5 to 20 for most clones with large constructs above 4000 bases^14^,^16^,^26^,^27^. An example of a hypoxia-sensitive reporter integrated into the vector system is shown in Fig. 2b. MSCs can react to hypoxic conditions, as found for example within tumors. Regulatory pathways induced by hypoxia can be mimicked in part by the treatment of cells with CoCl_2_, a chemical inducer of the hypoxia-inducible factor-1α (HIF1A) transcription factor^28^. CoCl_2_ titration of hypoxia (HIF1A) reporter-engineered MSCs show a dose-dependent activation of the reporter. In a second reporter example (Fig. 2c), the combination of an inducible transcription factor in the context of a WNT pathway reporter is shown^29^. hBMSCs were stably engineered using a pSBTR.WNT reporter (six TCF binding elements). A second pSBDEST vector introduced in parallel into the cells drives expression of TCF7 (a WNT transcription factor) under control of the inducible expression CMV/TO promoter (8476 bp, SB cassette 6620 bp). The canonical WNT pathway can be activated by recombinant human WNT3A, as shown here with a dose of 400 ng/ml, or upon treatment with (1 μg/ml) Doxycycline. Results between individual transfections of the same plasmid combinations were generally comparable, which is important for studies using primary hBMSCs which have a limited proliferative capacity in cell culture.

The reporter vectors can also be adapted to screen for more complex transcriptional features. Regulatory promoter modules are defined as a stretch of DNA, where two or more transcription factors conserved in orientation and distance act in concert to regulate gene transcription. Promoter modules can effectively integrate the downstream signals from multiple pathways and are thought to play an important role in tissue and signal specific gene transcription^30^. A previously validated promoter module comprised of NFkB (p65/p50 and p50/p50) elements with overlapping Sp1, nuclear factor of activated T cell (NFAT), krueppel like (KLFS) and myeloid zinc finger 1 (MZF1) elements was taken from the CCL5 promoter and cloned as a trimer into the pSBTR.reporter vector (see Supplemental Sequences). This module has been shown to react to a wide range of stimuli^31^. The pSBTR.3x (RaPM) reporter was strongly induced by stimulation with 15 ng/ml TNFα, with a more than additive effect seen when TNFα was combined with 45 ng/ml IFNγ (Fig. 2d).

The vector platform is also practical for characterization of downstream pathways activated in the context of cell co-culture studies (Fig. 3a). Myeloid cells produce factors that can activate target cells in a paracrine manner. Co-culturing engineered HEK293 reporter cells with unpolarized MCSF macrophages, or polarized M1/M2 macrophages leads to efficient activation of Hedgehog (GLI) and TGF-β (SMAD) signaling pathways, and to a lesser degree canonical WNT (TCF/LEF) and Hippo (TEAD) signaling^22^.

Signaling pathways are often assembled into higher order networks. This intertwining of pathways into coordinated regulatory networks allows cells to mount complex responses in response to a limited set of signals. It is now thought that a rather small number of signaling pathways, such as Hippo, TGF-β and WNT can account for a remarkable level of biological diversity during development, tissue homeostasis and repair. Hippo signaling is regulated at multiple levels including by nuclear translocation of the Hippo transcriptional co-activators YAP1 and TAZ (also known as WWTR1). This pathway controls organ size by regulating cell proliferation, apoptosis, and stem cell self-renewal^32^. Hippo signaling can converge with TGF-β and WNT signaling pathways in a context dependent manner to control cellular responses^33^. The vector system detailed is useful for characterizing aspects of crosstalk between regulatory pathways. This can be demonstrated by experiments employing pSBTR.reporter variants for Hippo (TEAD), TGF-β (SMAD), canonical WNT (TCF/LEF) and NFAT5 pathways, in combination with CMV/TO and pSBDEST driven expression of the transcriptional co-activators YAP1 (8462 bp, SB cassette 6606 bp) and TAZ (8270 bp, SB cassette 6414 bp). In HEK293 cells, doxycycline induced, constitutively active mutant YAP1 (S127A) or TAZ (S89A) efficiently activate Hippo (TEAD) signaling, but these highly selective events can also partially activate canonical WNT (TCF/LEF) and TGF-β (SMAD) signaling, while having no effect on NFAT5-responsive cells (Fig. 3b).

Osmotic stress can result in activation of the transcription factor NFAT5 which has been implicated as a general activator of genes linked to NaCl-based stress^34^. While NFAT5 plays a central role in this regard, other regulatory pathways may also be activated by increased NaCl. The vector system was used to monitor the effect of NaCl-based osmotic stress on NFAT5 signaling, and in parallel, the Hedgehog, TGF-β, and WNT pathways. Salt stimulation led to activation of the reporter constructs to varying degrees, showing that osmotic stress may have more widespread effects on transcription than previously anticipated (Fig. 3c)^35^.

Finally, the reporter vectors are amenable for inhibitor studies as exemplified by activating protein 1 (AP1) reporter HEK293 cells stimulated with recombinant human EGF protein^36^, and inhibited with the MAPK inhibitor PD98059 or PI3K inhibitor Wortmannin. PD98059 significantly reduces the AP1 activation seen with EGF stimulation, while Wortmannin was found to have no effect (Fig. 3d).

From start to finish, vector construction can be completed in days to weeks. When used in concert with SB transposase and nucleofection techniques, the resultant vectors can be efficiently incorporated into the genomes of primary or immortalized cells. The vector platform described here allows characterization of diverse signaling processes in a large number of research fields ranging from the monitoring of changes in homeostatic microenvironments by inflammatory cells, to drug effects in cancer. The system is amenable for high throughput studies involving cloning, stable transfection and screening, and thus can be used for the generation of large datasets in relatively short timeframes.

## Additional Information

**How to cite this article**: Jäckel, C. *et al*. A vector platform for the rapid and efficient engineering of stable complex transgenes. *Sci. Rep*. **6**, 34365; doi: 10.1038/srep34365 (2016).

## Supplementary Material

Supplementary Information

## Figures and Tables

**Table 1:** Summary of the vectors presented here.

Promoterless, SB-compatible Gateway destination vectors			Addgene Plasmid
pSBDEST.B	Blasticidin selection	7800 bp	#79460
pSBDEST.H	Hygromycin selection	8419 bp	#79464
pSBDEST.N	G418 selection	8212 bp	#79468
pSBDEST.Z	Zeocin selection	7748 bp	#79470
pSBDEST.IP	IRES tied to Puromycin selection	7294 bp	#79472
**SB-compatible** ***Gaussia*** **luciferase reporters, all with TET-Repressor expression and Blasticidin selection**			**Addgene Plasmid**
pSBTR.AP1	CMVMin driven FOS JUN reporter	8465 bp	#79475
pSBTR.Hedgehog	CMVMin driven GLI1 reporter	8490 bp	#79477
pSBTR.Hippo	CMVMin driven TEAD reporter	8585 bp	#79478
pSBTR.Hypoxia	MiniTK driven HIF1A reporter	8582 bp	#79479
pSBTR.LXR	CMVMin driven NR1H2/3 reporter	8459 bp	#79480
pSBTR.NFAT5	CMVMin driven NFAT5 reporter	8446 bp	#79481
pSBTR.WNT	CMVMin driven TCF reporter	8588 bp	#79482
pSBTR.TGF	CMVMin driven SMAD/TGFβ reporter	8428 bp	#79483
pSBTR.3x (RaPM)	CMVMin driven CCL5 promoter module reporter	8492 bp	#79484

**Table 2:** Compatible Entry clones.

Compatible 2-fragment MultiSite Gateway entry clones, first fragment (attL1, attR5)			Addgene Plasmid
pENTR221-CMV/TO.Pro	CMV/TO promoter	3267 bp	#79485
pENTR221-CMV.Pro	CMV promoter	3246 bp	#79486
pENTR221-RSV.Pro	RSV promoter	3855 bp	#79487
pENTR221-EF1.Pro	Human EF1 promoter	2867 bp	#79488
pENTR221-PGK.Pro	Human PGK promoter	3142 bp	#79489
pENTR221-CAG.Pro	CAG promoter	4238 bp	#79490
pENTR221-IL6.Pro	Human IL6 promoter	4250 bp	#79491
pENTR221-CCL5.Pro	CCL5/RANTES promoter	3640 bp	#79492
pENTR221-VEGF.Pro	Mouse VEGF promoter	5119 bp	#79493
**Compatible 2-fragment MultiSite Gateway entry clones, second fragment (attL5, attL2)**			**Addgene Plasmid**
pENTR221-Gaussia	*Gaussia* Luciferase CDS	3132 bp	#79494
pENTR221-Firefly	Firefly Luciferase CDS	4392 bp	#79495
pENTR221-Renilla	*Renilla* Luciferase CDS	3980 bp	#79496
pENTR221-GLI1	Human GLI1 CDS	5934 bp	#79497
pENTR221-TCF7	Human TCF7 CDS	4146 bp	#79498
pENTR221-WNT1	Human WNT1 CDS	3678 bp	#79499
pENTR221-WNT3	Human WNT3 CDS	3625 bp	#79500
pENTR221-WNT3A	Human WNT3A CDS	3619 bp	#79501
pENTR221-YAP1 CA	Human YAP1 S127A mutant CDS	4132 bp	#79502
pENTR221-YAP1	Human YAP1 CDS	4054 bp	#79503
pENTR221-TAZ CA	Human TAZ S89A mutant CDS	3940 bp	#79504
pENTR221-TAZ	Human TAZ CDS	3940 bp	#79505
pENTR221-NFAT5	Human NFAT5 CDS	7424 bp	#79506
pENTR221-SMAD3	Human SMAD3 CDS	4008 bp	#79507
pENTR221-sGFP	Super GFP CDS	3339 bp	#79508
pENTR221-mCherry	mCherry RFP CDS	3320 bp	#79509
pENTR221-Tomato	Tomato RFP CDS	4048 bp	#79510
pENTR221-FOS	Human FOS CDS	3700 bp	#79511
pENTR221-JUN	Human JUN CDS	3653 bp	#79512
pENTR221-NR1H2	Human NR1H2 CDS	3943 bp	#79513
pENTR221-NR1H3	Human NR1H3 CDS	3766 bp	#79514
pENTR221-HIF1A CA	Human HIF1A (P402A, P564A) mutant CDS	5437 bp	#79515
