# Supplementary material for: A vector platform for the rapid and efficient engineering of stable complex transgenes
Source: Sci Rep. 2016 Oct 3;6:34365. doi: 10.1038/srep34365 (PMC5046065; doi:10.1038/srep34365)
Supplement: Supplementary Information [file srep34365-s1.pdf]

A vector platform for the rapid and efficient engineering of stable complex transgenes

Carsten Jäckel[1], Melanie Schmitt-Nogueira[1], Nadja Ehni[1], Christiane Kraus[1], Julius Ranke[1], Maike Dohmann[1], Elfriede Noessner[2] and Peter J. Nelson[1*]

[1]Medizinische Klinik und Poliklinik IV, Klinikum Innenstadt, Ludwig-Maximilians-University Munich, Clinical Biochemistry Group, Munich, Germany

[2]Core Facility Immunanalytik, Helmholtz Zentrum, Großhadern, Germany

Corresponding author: [*]Peter J. Nelson, Ph.D. Medizinische Klinik und Poliklinik IV, Klinikum Innenstadt, Ludwig-Maximilians-University Munich, Nephrology Center, Clinical Biochemistry Group, Schillerstrasse 42, 80336, Munich, Germany

Supplemental Figures:

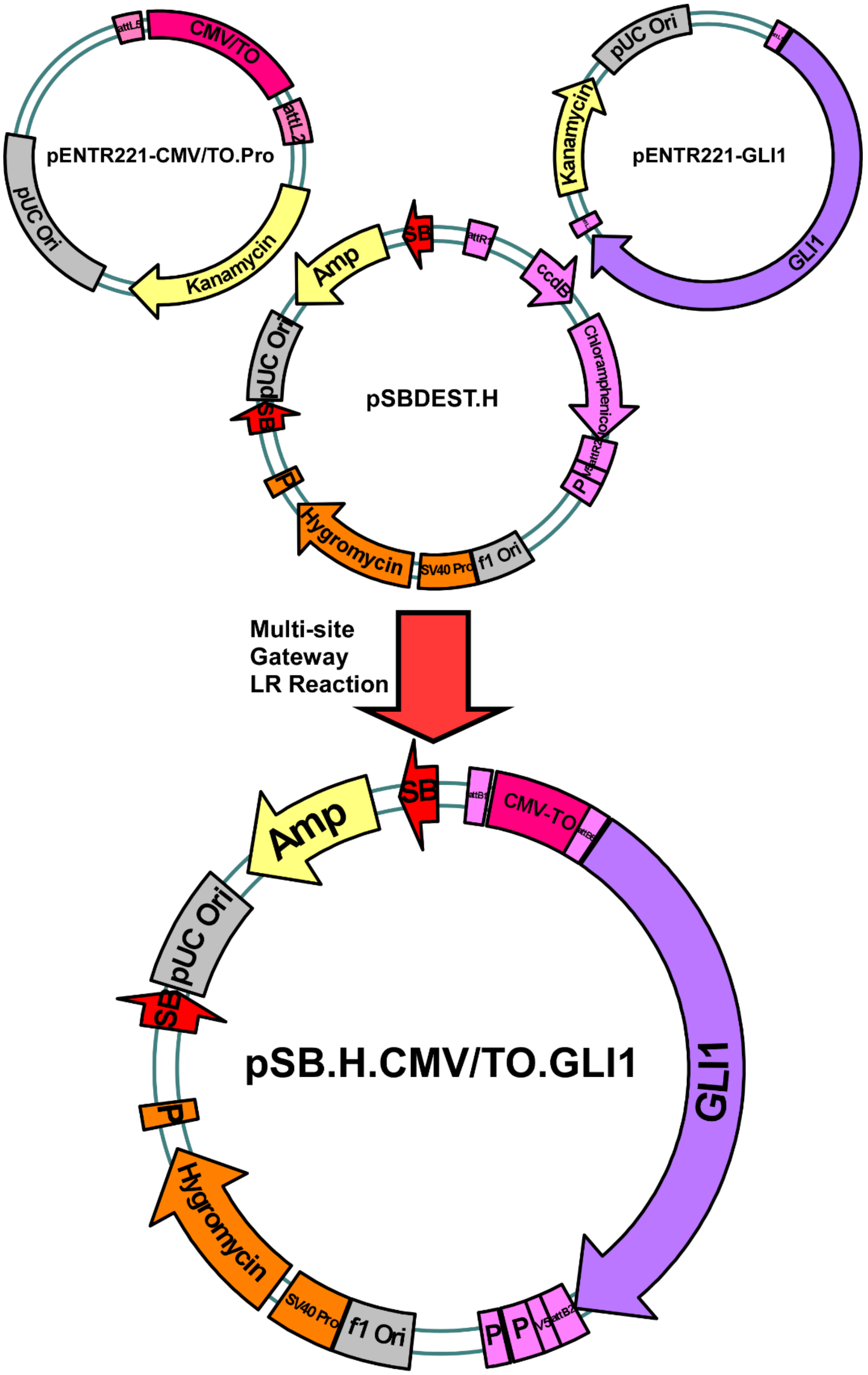

Supplemental Figure S1. Sample MultiSite Gateway reaction. MultiSite Gateway reactions can be used to generate complex, SB-compatible vectors in a rapid, site-specific process. Here, an inducible expression vector for GLI1 is generated from two Entry clones in the pSBDEST.H vector.

Supplemental Sequences:

Minimal CMV Promoter:

CGAGATCTAGACTCTAGAGGGTATATAATGGAAGCT

Minimal TK Promoter:

TTCGCATATTAAGGTGACGCGTGTGGCCTCGAACACCGAGCGACCCTGCAGCGAC
CCGCT

TAA

AP1 Binding element (6x multimer):

TGACTCA, Linker Sequence: TCAAGCA

GLI binding element (8x multimer):

GACCACCCA

TEAD binding element (8x multimer):

ACATTCC, varying linker sequences

HIF binding element (3x multimer)

GTGCAGGACGTGACAAA

LXR binding element (3x multimer)

TGACCAGCAGTAACC

NFAT5 binding element (dimer)

CAGCGGTAATTTTCCACCA

TCF/LEF binding element (12x multimer)

ATCAAAG

SMAD binding element (5x multimer)

AGCCAGACAGT

RaPM element (3x multimer)

CTATTTTGGAAACTCCCCTTAGGGGATGCCCCTC, Linker sequence: AACTGCTCGAG
